# Editorial: The role of circular RNAs and MicroRNAs in non-small cell lung cancer: from mechanisms to therapeutic potential

**DOI:** 10.3389/fonc.2026.1873294

**Published:** 2026-05-27

**Authors:** Alexandru Tirpe, Paola Campomenosi, Ioana Berindan-Neagoe

**Affiliations:** 1 Department of Genomics, MEDFUTURE Institute for Biomedical Research, Iuliu Hațieganu University of Medicine and Pharmacy, Cluj-Napoca, Romania; 2 Universita degli Studi dell’Insubria Dipartimento di Biotecnologie e Scienze della Vita, Varese, Italy; 3 Doctoral School, Iuliu Hațieganu University of Medicine and Pharmacy, Cluj-Napoca, Romania; 4 The Academy of Medical Sciences, Bucharest, Romania

**Keywords:** circRNA, editorial, microRNA, ncRNA, NSCLC, lung cancer

When referring to the GLOBOCAN 2022 statistics (1), lung cancer is estimated to be the leading malignancy worldwide in terms of both incidence and mortality, with approximately 2.48 million new cases and 1.81 million deaths reported in 2022 alone. Over the past decades, continued research efforts have significantly improved patient outcomes, driven in part by the introduction of immunotherapy and targeted therapies that exploit specific molecular drivers of the disease.

Non-coding RNAs represent a class of functional molecules that are largely involved in key oncogenic processes, ranging from epithelial-to-mesenchymal transition (EMT) and invasion, to metabolic reprogramming, angiogenesis and therapy resistance (2).

The present Research Topic, entitled “The Role of Circular RNAs and MicroRNAs in Non-Small Cell Lung Cancer: From Mechanisms to Therapeutic Potential” aims to advance the understanding of the roles of microRNAs (miRNAs, miRs) and circular RNAs (circRNAs) in non-small cell lung cancer (NSCLC), the most prevalent subtype of lung cancer (1).

Li et al. (3) investigated the oncogenic role of circUBE2G1 in lung adenocarcinoma (LUAD), a subtype of NSCLC, while focusing specifically on its contribution to lymph node (LN) metastasis. The authors found that circUBE2G1 is upregulated in LUAD tissues, where it is positively associated with LN status as well as with poorer overall and disease-free survival in LUAD patients. From a functional standpoint, circUBE2G1 promoted LUAD lymphangiogenesis *in vitro* and facilitated LUAD LN metastasis *in vivo*. Furthermore, this study revealed that circUBE2G1 directly interacts with hnRNPU in LUAD cells. CircUBE2G1 recruits hnRNPU to enhance H3K27ac at the VEGF-C promoter, leading to transcriptional activation of VEGF-C. Overall, the authors identified a key pro-oncogenic function of circUBE2G1 and highlighted it as a promising therapeutic target in LUAD (3).

Tan et al. (4) studied the role of hsa_circ_0001859 in NSCLC, providing the first comprehensive evidence for the association of the circ_0001859/miR-101-3p/MMP1 oncogenic axis in this malignancy. The authors demonstrated that circ_0001859 is significantly upregulated in both NSCLC tissues and cell lines, with a strong correlation with advanced TNM stage and lymph node metastasis. From a functional standpoint, circ_0001859 silencing led to inhibitory effects on cell proliferation, migration, and invasion *in vitro*, as well as reduced tumor volume and weight in *in vivo*/xenograft models. The primary novelty of the study lies in the detailed characterization of the circ_0001859/miR-101-3p/MMP1 regulatory axis using dual-luciferase reporter and RNA immunoprecipitation assays. Circ_0001859 sponges miR-101-3p, thereby modulating MMP1 levels. The authors concluded that circ_0001859 is involved in regulating NSCLC growth and metastasis via the axis, and highlighted it as a potential diagnostic biomarker and therapeutic target (4).

Førde et al. (5) comprehensively assessed the prognostic and functional roles of two microRNAs — miR-17-5p, and miR-20a-5p — in a large cohort of 553 consecutively resected NSCLC patients using *in situ* hybridization and digital image analysis within the tumor compartment. High miR-20a-5p expression emerged as a predictor of favorable disease-specific survival, particularly in patients with LUSC and node-positive (N+) cases. Concomitantly, increased miR-17-5p expression was associated with poor prognosis in node-negative (N0) patients. Functional assays showed that overexpression of both miR-20a-5p and miR-17-5p increased invasion and migration in A549 and H520 cell lines. The authors conclude that the prognostic role of miR-20a-5p and miR-17-5p in early-stage NSCLC is context-dependent, and hypothesize that miR-17-5p is associated with early invasive transformation, while miR-20a-5p may exert tumor-suppressive functions under certain conditions (5).

Wei et al. (6) provide a critical review of miR-155 in NSCLC, presenting the various roles of microRNAs in cancer and specifically in NSCLC, such as miR-21, miR-126, and miR-155. The paper details the miR-155-dependent mechanisms that modulate key biological processes, such as cancer cell proliferation, migration, invasion, and metastasis, as well as its role in chemotherapy resistance and immune response. The authors also analyze the potential clinical applications of miR-155, together with the delivery strategies of anti-miR-155 agents. Furthermore, Wei et al. provide a comprehensive list of validated miR-155 targets in NSCLC, along with their associated biological functions. The novelty of this paper lies in its up-to-date integration of molecular mechanisms with potential clinical applications, positioning miR-155 as a candidate diagnostic biomarker in NSCLC (6).

Tirpe et al. (2) provided a comprehensive review that bridges the gap between the coding mutational landscape and non-coding RNA regulation in NSCLC. The authors mapped essential driver mutations and molecular alterations, including EGFR, ALK, ROS1, RET, MET, NTRK, as well as BRAF and KRAS to their downstream signaling cascades, while providing critical information on current targeted therapeutics in this context. The review demonstrates how specific miRNAs act as key modulators of these pathways, and categorizes them based on their roles in core cancer progression processes, ranging from survival and invasion, to neoangiogenesis, metabolic reprogramming and therapy resistance. The primary novelty of this paper lies in its integrative and translational perspective, providing an updated atlas of the miRNA-mutation landscape. The authors synthesize clinical data with preclinical findings, highlighting miRNAs as potential biomarkers for diagnosis and prognosis, as well as for potential therapeutic interventions. Overall, the paper serves as a valuable roadmap for navigating the complex molecular framework of NSCLC (2).

The present Research Topic underlines the intricate mechanisms of non-coding RNAs, specifically miRNAs and circRNAs, in NSCLC progression. The findings presented herein range from complex signaling pathways, such as the involvement of circUBE2G1/hnRNPU/VEGF-C axis as a pro-oncogenic entity in LUAD, to comprehensive review papers addressing miR-155 and the mutational landscape, as well as miRNA involvement in NSCLC.
